# Comparison the clinical outcomes of intravenous and subcutaneous erythropoietin administration in hemodialysis patients of plateau regions

**DOI:** 10.1186/s12882-026-04790-8

**Published:** 2026-03-04

**Authors:** Yao Li, Chuanxiong Yang, Jing Xu, Dan Li, Minghong Xu, Zhu Zhou

**Affiliations:** 1 Department of Nephrology, First Affiliated Hospital, Kunming Medical University, Kunming, Yunnan Province China; 2 Department of Clinical Nutrition, The Second People’s Hospital of Kunming, Kunming, China; 3 Department of Urology, Second Affiliated Hospital, Kunming Medical University, Kunming, Yunnan Province China

**Keywords:** Hemodialysis, Erythropoietin, Epoetin (EPO), Administration, Anemia

## Abstract

**Background:**

Recombinant human erythropoietin (epoetin) is the mainstay of anemia management in hemodialysis patients. Intravenous and subcutaneous routes are the most commonly used administration methods. While differences in pharmacokinetics and efficacy between the two routes have been reported, limited data are available regarding patients residing in high-altitude regions, where chronic hypoxia may alter erythropoiesis. Therefore, we compared the required epoetin dose and the incidence of adverse events of intravenous and subcutaneous administration in hemodialysis patients in the southwestern China plateau region.

**Methods:**

We conducted a multicenter, retrospective cohort study including 5896 adult patients between January 1, 2023 and December 31, 2023. After ensuring no statistical differences in baseline data through propensity score matching, we compared the epoetin dosage and adverse event incidence between the two administration routes (intravenous and subcutaneous) during one year follow-up period.

**Results:**

The intravenous group required higher epoetin doses. In the short term, the intravenous group achieved a higher rate of hemoglobin attainment (Hb ≥ 110 g/L) than the subcutaneous group, but rates were comparable at one year. Serum potassium was lower in the subcutaneous group within one year. Adverse event incidence and hemoglobin level were similar between the two groups.

**Conclusions:**

For hemodialysis patients in the southwestern plateau regions of China, subcutaneous and intravenous epoetin administration show comparable efficacy, with subcutaneous therapy being more dose-efficient and potentially more practical in clinical use.

**Clinical trial number:**

Clinical trial number is not applicable.

## Introduction

Anemia is one of the most prevalent complications in patients with end-stage renal disease (ESRD) receiving maintenance hemodialysis (HD). It is strongly associated with increased cardiovascular morbidity, reduced quality of life, and higher mortality rates [1]. The introduction of erythropoiesis-stimulating agents (ESAs), such as erythropoietin (EPO), has significantly improved anemia management in this population [2].

EPO can be administered either intravenously (IV) during dialysis sessions or subcutaneously (SC) after dialysis [3, 4]. Previous studies have suggested that SC administration may achieve comparable hemoglobin targets at lower doses, owing to slower absorption and prolonged half-life [5]. And randomized clinical trials in patients with renal failure have demonstrated that higher doses of EPO are associated with an increased risk of cardiovascular events and mortality [6].

Patients residing in high-altitude regions, such as southwestern China, are chronically exposed to persistent hypobaric hypoxia, which may alter their endogenous hematopoietic function. These physiological adaptation mechanisms theoretically influence the pharmacokinetic and pharmacodynamic properties of exogenous EPO, thereby affecting dosage requirements and treatment safety [7, 8]. However, existing evidence remains insufficient regarding the comparative efficacy and clinical outcomes of IV versus SC administration of EPO in this specific population, leaving the optimal treatment regimen yet to be determined. To provide more clinical evidence for hemodialysis patients in high-altitude regions, we compared the efficacy (EPO dose and hemoglobin level) and safety (adverse events) of IV versus SC administration of EPO in HD patients residing in the southwestern China plateau.

## Methods

### Study design and population

This retrospective study, conducted from January 1, 2023, to December 31, 2023, included 8799 hemodialysis patients in the southwestern China plateau region. Inclusion criteria were: age ≥ 18 years; receipt of regular hemodialysis; availability of complete clinical and laboratory records. Exclusion criteria were: missing key clinical or laboratory data greater than 10% (201); uncertain or undocumented outcome events (89); no information available regarding EPO treatment, dosage, route of administration (367) or received both administration routes (26). And the remaining 8116 patients were categorized into IV group (5162 ) and SC group (2954). After employing propensity score matching (PSM), among 59 dialysis centers, 2948 patients were enrolled in the IV and SC groups respectively. All patients received standardized treatment according to the EPO instructions, with EPO dosage adjusted promptly based on hemoglobin levels.

### Data collection

Demographic and clinical data included: age, gender, body mass index (BMI), dialysis duration, history of cardiovascular and cerebrovascular disease, serum albumin and potassium, EPO dose (IU/kg/week). Hematological and biochemical parameters: hemoglobin (Hb), hematocrit (Hct), ferritin, transferrin saturation (TSAT). Adverse events were death and cardiovascular and cerebrovascular events. Data collection occurs every four months, with a total follow-up period of one year.

### Outcomes

The primary outcome of the study was hemoglobin levels and the dose of EPO administered, while the secondary outcome was the occurrence of adverse events.

### Statistical analysis

To minimize potential confounding, propensity score matching (PSM) was performed. Propensity scores were estimated using a multivariable logistic regression model with baseline covariates including age, sex, history of disease, dialysis duration, hemoglobin, albumin, and serum potassium, et al. A 1:1 nearest-neighbor matching algorithm without replacement was applied. A caliper width of 0.2 times the standard deviation of the logit of the propensity score was used to restrict matches and reduce the risk of poor-quality matching. After matching, covariate balance between the two groups was assessed using standardized mean differences (SMD), with an SMD < 0.1 indicating acceptable balance. Continuous variables were expressed as mean ± standard deviation or median (interquartile range) and compared using paired t-test or paired Wilcoxon signed-rank test. Categorical variables were summarized as counts and percentages and compared using the McNemar test and Bowker test. Cox proportional hazards regression analysis was performed to evaluate survival-related outcomes. P-value < 0.05 was considered statistically significant.

## Results

### Clinical characteristics of patients

**Fig. 1:**
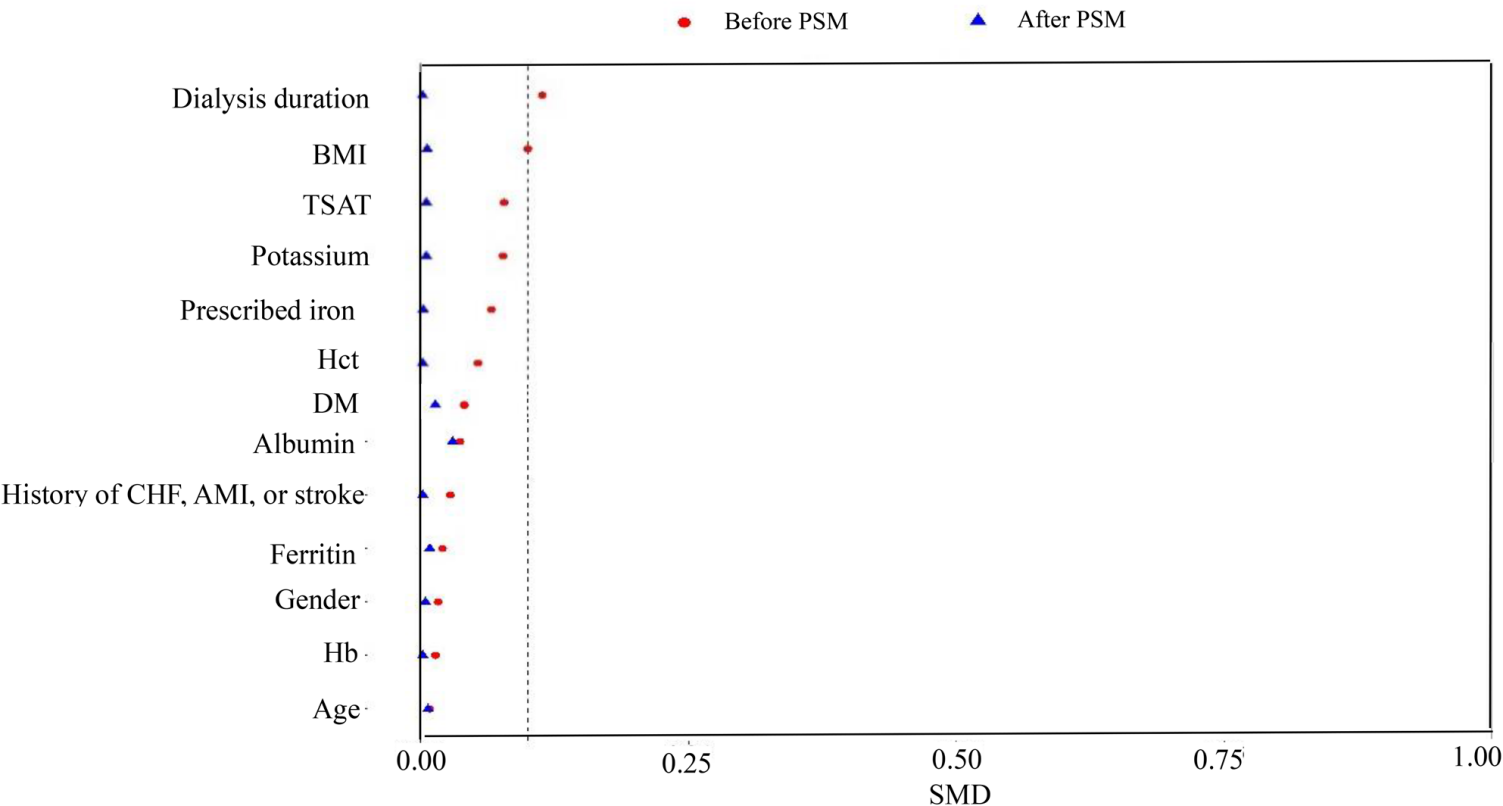
SMD of covariates before and after PSM. BMI: body mass index, Hb: hemoglobin, Hct: hematocrit, TSAT: transferrin saturation, CHF: congestive heart failure, AMI: acute myocardial infarction, DM:diabetes mellitus

After PSM to minimize the differences between the IV and SC groups, 5896 hemodialysis patients were finally included. After PSM, the average age of patients was (51.80 ± 14.35) years, and their BMI was (20.75 ± 4.40) kg/m^2^. The data for the two groups before and after PSM are detailed in Table 1. The SMD of covariates before and after PSM is shown in Fig. 1. The two groups showed no differences in age, BMI, gender composition, history of previous diseases, hemoglobin levels, or iron metabolism-related indicators.

**Table 1:** Baseline characteristics of patients before and after PSM

Variable	Before PSM				After PSM			
	IV (*n* = 5162)	SC (*n* = 2954)	*P*	SMD	IV (*n* = 2948)	SC (*n* = 2948)	*P*	SMD
Age (years)	51.74 ± 14.36	51.85 ± 14.54	0.729	0.008	51.75 ± 14.16	51.85 ± 14.53	0.792	0.007
Male/Female	3103/2059	1824/1130	0.147	0.034	1806/1142	1819/1129	0.728	0.009
BMI (kg/m^2^)	21.21 ± 4.39	20.76 ± 4.46	<.001	0.100	20.74 ± 4.34	20.77 ± 4.46	0.816	0.006
Hb(g/L)	105.80 ± 17.12	105.56 ± 17.07	0.547	0.014	105.57 ± 17.33	105.54 ± 17.06	0.939	0.002
Hct (%)	33.32 ± 7.18	33.05 ± 4.98	0.074	0.054	33.07 ± 4.96	33.08 ± 4.85	0.940	0.002
TSAT (%)	38.74 ± 14.13	37.97 ± 9.93	0.004	0.078	37.87 ± 10.60	37.92 ± 9.60	0.841	0.006
Ferritin (ug/L)	146.29 ± 246.71	150.36 ± 199.71	0.418	0.020	148.88 ± 242.42	150.57 ± 199.11	0.770	0.008
Albumin (g/L)	39.14 ± 6.35	38.78 ± 9.90	0.045	0.037	38.97 ± 5.88	38.67 ± 7.56	0.094	0.039
Potassium (mmol/L)	5.01 ± 0.61	4.97 ± 0.60	0.001	0.077	4.96 ± 0.61	4.97 ± 0.59	0.844	0.005
Dialysis duration (month)	7.00(2.00,25.00)	7.00(2.00,22.00)	0.195	0.113	6.00(2.00,20.00)	7.00(2.00,22.00)	0.038	0.002
< 6 m n (%)	2284(44.3)	1316(44.6)	0.007	0.006	1390(47.2)	1310(44.4)	0.058	0.056
6–12 m n (%)	709(13.7)	470(15.9)		0.063	414(14)	470(15.9)		0.053
12–24 m n (%)	826(16.0)	482(16.3)		0.008	499(16.9)	482(16.4)		0.013
≥24m n (%)	1343(26.0)	686(23.2)		0.065	645(21.9)	686(23.3)		0.033
History of CHF, AMI, or stroke n (%)	502 (9.72)	206 (6.97)	<.001	0.108	212 (7.19)	206 (6.99)	0.761	0.008
DM n (%)	893 (17.30)	388 (13.13)	<.001	0.123	375 (12.72)	388 (13.16)	0.614	0.013
Prescribed iron n (%)	1594 (30.88)	1107 (37.47)	<.001	0.136	1095 (37.14)	1102 (37.38)	0.850	0.005

BMI: body mass index, Hb: hemoglobin, Hct: hematocrit, TSAT: transferrin saturation, CHF: congestive heart failure, AMI: acute myocardial infarction, DM: diabetes mellitus

### Differences in hb levels and EPO dosing between SC and IV groups

**Fig. 2:**
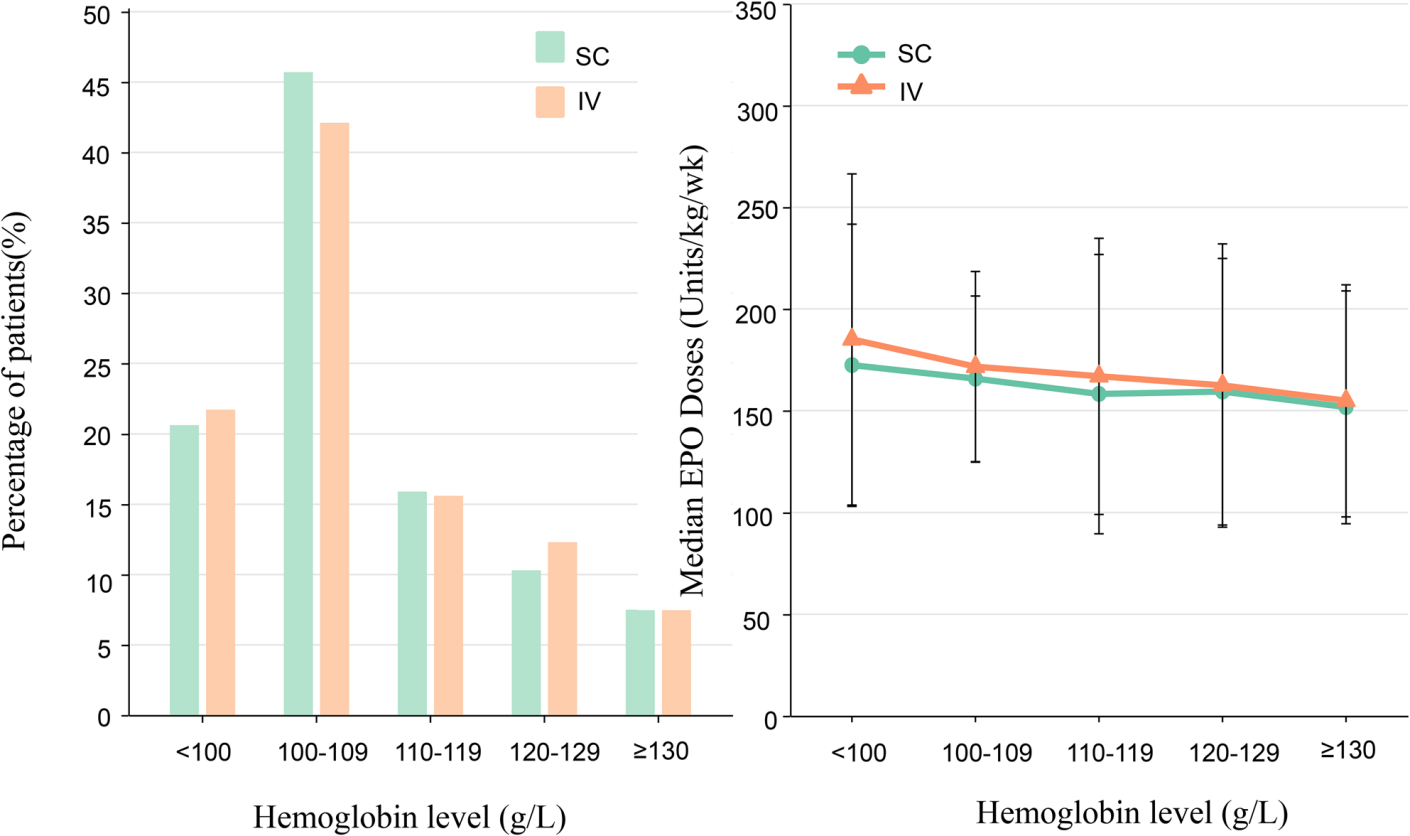
**a**: The proportion of patients receiving SC or IV EPO therapy whose hemoglobin levels were maintained within different ranges (< 100 to ≥ 130 g/L). **b**: The required EPO dose (units/kg/week) of SC or IV group to achieve different hemoglobin levels (< 100 to ≥ 130 g/L)

The mean Hb level in the SC group within one year of follow-up was (108.37 ± 15.29) g/L, while that in the IV group was (108.79 ± 15.82) g/L. There was no difference between the two groups (t = 1.045, *P* = 0.296, 95% CI for the difference: -1.20―0.37). The proportion of patients maintaining Hb levels within different ranges (< 100 to ≥ 130 g/L) showed no difference between the IV and SC groups (χ² = 17.13, *P* = 0.072) (Fig. 2a). The mean Hct level in the SC group within one year was (37.42 ± 5.44) %, while that in the IV group was (37.33 ± 5.38) %, (t = 0.666, *P* = 505, 95% CI for the difference: -0.18―0.37). The mean EPO doses in the SC group was (163.88 ± 56.71) units/kg/week, while that in the IV group was (171.24 ± 63.33) units/kg/week (t = 4.643, *P* < 0.001, 95% CI for the difference: 4.25―10.46). Analysis of EPO dosing across different Hb levels revealed that in the range of < 100 and 100–109 g/L, the SC group received lower EPO doses than the IV group (*P* < 0.05) (Fig. 2b).

**Fig. 3:**
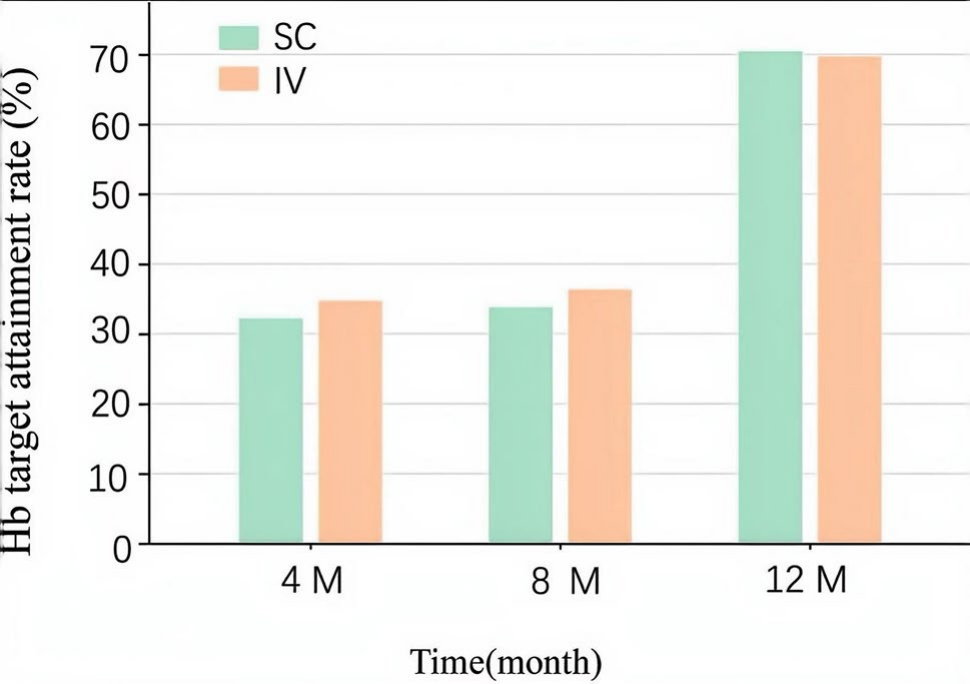
Hemoglobin target attainment rates for the two groups at different time

After 4 months and 8 months, the IV group achieved higher hemoglobin target(Hb>110 g/L) rates than the SC group (*P*<0.001). However, after 12 months of follow-up, hemoglobin target rates significantly increased in both groups with no difference observed (Fig. 3).

### Adverse clinical outcomes

The incidence of adverse events in the study cohort was statistically analyzed. Among the 5,896 study participants, 280 (4.8%) patients died within one year of follow-up. The primary cause of death was cardiovascular events, accounting for 43.2%, while cerebrovascular events constituted 11.4%. Among cardiovascular events, congestive heart failure (CHF) accounted for 65.6%, while acute myocardial infarction (AMI) accounted for 12.3%. Conditional logistic regression analysis demonstrated that the route of EPO administration (SC and IV) was not significantly associated with the occurrence of adverse events, including all-cause mortality, cardiovascular events, and cerebrovascular events (*P* > 0.05).

**Fig. 4:**
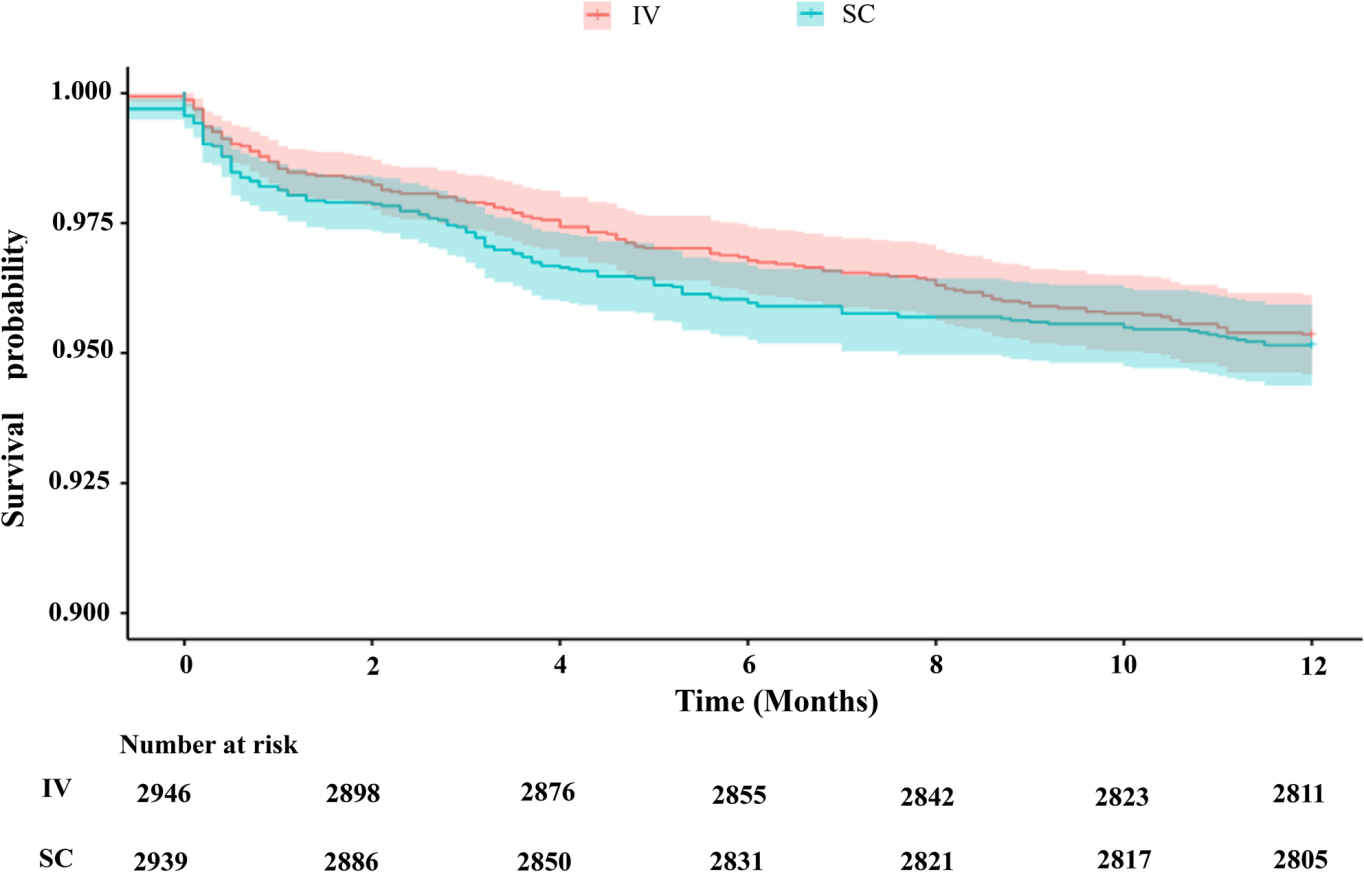
Kaplan–Meier survival curves comparing the IV and SC administration groups

To further assess time-to-event outcomes, Cox proportional hazards regression was performed. In univariate analysis, SC administration was not associated with adverse outcomes compared with IV administration (HR 0.94, 95% CI 0.75–1.18, *P*=0.621). This finding remained unchanged in multivariable models adjusted for baseline characteristics (HR 0.99, 95% CI 0.79–1.25, *P*=0.961). Significant predictors of adverse events included older age, longer dialysis duration, history of cerebrovascular or cardiovascular disease and combined DM. Other parameters, such as baseline hemoglobin level, EPO doses, were not independently associated with adverse outcomes (Table 2). Kaplan–Meier survival analysis showed that the survival curves for the IV and SC groups remained largely overlapping during the 12-month follow-up. Survival rates in both groups was similar. (Fig. 4). The mean serum potassium in the SC group within one year of follow-up was (4.89±0.57) mmol/L, while that in the IV group was (4.94±0.56) mmol/L (t=3.242, *P*=0.001, 95% CI for the difference:0.02―0.08).

**Table 2:** Univariate and multivariate Cox proportional hazards model analysis of adverse event

Variables	Univariate analysis			Multivariate analysis		
	β	*P*	HR(95%CI)	β	*P*	HR(95%CI)
Previous AMI, CHF, or stroke	0.51	0.009	1.67 (1.14–2.44)	0.40	0.040	1.49(1.02–2.17)
Age	0.05	< 0.001	1.05 (1.04–1.06)	0.05	< 0.001	1.05 (1.04–1.06)
Dialysis duration	0.01	< 0.001	1.01 (1.01–1.02)	0.02	< 0.001	1.02 (1.01–1.02)
SC versus IV	-0.06	0.621	0.94 (0.75–1.18)	-0.01	0.961	0.99 (0.79–1.25)
BMI	-0.02	0.088	0.98 (0.95-1.00)	-0.02	0.076	0.98 (0.95-1.00)
Hb	0.00	0.632	1.00 (0.99–1.01)	0.00	0.475	1.00 (1.00-1.01)
EPO doses	0.00	0.115	1.00 (1.00–1.00)	0.00	0.055	1.00 (1.00–1.00)
DM	0.46	0.002	1.59 (1.19-2.13)	0.36	0.019	1.43 (1.06-1.96)

BMI: body mass index, Hb: hemoglobin, Hct: hematocrit, TSAT: transferrin saturation, CHF: congestive heart failure, AMI: acute myocardial infarction, DM: diabetes mellitus

## Discussion

In the present study of HD patients from a high-altitude region, we compared the efficacy and safety of IV and SC administration of EPO. We found that mean Hb levels during one year of follow-up were similar between the two groups, although IV administration required higher doses. There was no difference in adverse events between the IV and SC groups, suggesting that both administration methods are viable options.

Previous clinical studies have consistently shown that SC can maintain hemoglobin targets with 25–40% lower weekly doses compared with IV administration [9]. Our study found that although the overall dose in the SC group was lower than that in the IV group, the absolute and relative differences were small (approximately 7.36 units/kg/week, or about 4.3%). Possible explanations include: First, patients in a plateau chronic hypoxia setting exhibit a degree of endogenous hematopoietic stimulation and microcirculatory adaptation, which may mitigate dose differences introduced by exogenous administration routes [10, 11]. Second, multicenter real-world treatment strategies (dosing frequency, dose adjustment thresholds) differ from research conditions, diluting overall dose disparities. Our study also found that the rate of achieving target hemoglobin levels was higher in the IV group than in the SC group in the short term, but the two groups were similar after one year of follow-up. This can be explained by demonstrated pharmacokinetic differences between the two administration routes [9, 10]. IV administration results in a rapid surge in serum EPO concentration, followed by swift clearance, while SC administration takes effect slowly but provides long-lasting effects.

Although previous studies have suggested that EPO therapy may inhibit potassium clearance during dialysis, the mechanism is typically attributed to increased hematocrit and the consequent reduction in dialyser solute clearance rate [12]. In our cohort, hematocrit levels were comparable between the IV and SC groups, making it unlikely that reduced dialyser potassium clearance due to hematocrit could explain the observed differences. Instead, these data align more closely with a scenario where the SC group experienced a slight decline in potassium levels over one year, while the IV group remained largely stable. This may relate to EPO’s regulation of potassium transport across cell membranes by influencing ion channels and transporters [13–15]. Differences in the bioavailability and dosage of EPO of SC and IV group in our study could account for the potassium variations. However, serum potassium levels are influenced not only by EPO therapy but also by multiple factors including dialysis parameters, potassium concentration in dialysate, use of renin-angiotensin-aldosterone system (RAAS) inhibitors or diuretics, nutritional intake, and residual renal function [16–18]. Since these variables were not systematically evaluated in our analysis, a definitive causal relationship between EPO administration route and serum potassium levels cannot yet be established. Future prospective studies with more comprehensive control of potential confounding factors are needed to further validate this finding.

Our findings indicate no significant difference in adverse event rates between IV and SC administration, suggesting both routes are generally safe. This observation implies that clinical decisions regarding EPO administration routes may be guided by factors such as convenience, patient compliance, and cost [19]. Importantly, our Cox regression analysis identified advanced age, prolonged dialysis duration, and a history of cardiovascular or cerebrovascular disease as independent risk factors for adverse outcomes, consistent with prior studies [20–22]. These findings underscore that patient characteristics typically exert the greatest influence on adverse event rates. Mechanistically, elderly patients and patients with longer dialysis duration experience vascular calcification, inflammation, and malnutrition, which may diminish the protective effects of EPO therapy [23]. Notably, the overall mortality observed in our cohort was lower than reported in many previous dialysis studies. Several factors may account for this finding. First, a large proportion of our patients had a dialysis duration of less than six months, representing a relatively early stage of renal replacement therapy, during which the cumulative burden of dialysis-related complications has not fully developed. Second, the follow-up period of one year, while sufficient to assess hematological responses, may not capture later-onset cardiovascular or cerebrovascular events that significantly contribute to long-term mortality in dialysis populations [24, 25]. Third, our cohort is drawn from a high-altitude region, where chronic hypoxia induces adaptive physiological changes. These include sustained endogenous EPO production, enhanced microvascular perfusion, increased capillary density, and improved oxygen delivery to tissues, which together may confer a degree of cardiovascular and hematological resilience [26, 27]. Such high-altitude adaptations may partially influence the risk of adverse events and could be one of several factors contributing to the relatively lower mortality observed in this cohort.

It should be acknowledged that this study has several limitations. First, the observational and retrospective design limits causal inference. Second, the relatively short one-year follow-up period may not fully capture late-onset cardiovascular or cerebrovascular events, which are important contributors to long-term morbidity and mortality in the dialysis population. Third, our cohort is geographically specific, originating from a high-altitude region, which may limit the generalizability of findings to other populations. Future prospective studies with longer follow-up periods, more comprehensive data collection, and careful adjustment for confounding factors are necessary to validate and extend these observations.

## Conclusion

In conclusion, both SC and IV administration of EPO achieve similar long-term hemoglobin targets and safety in hemodialysis patients at high altitude, with SC administration achieved comparable outcomes with lower EPO use. Adverse events were mainly determined by patient factors such as age, dialysis vintage, and cardiovascular history, rather than administration route. These findings highlight the importance of individualized anemia management.

## Data Availability

Data is available on request from the authors. The data supporting this study’s findings are available from the corresponding author Zhou Zhu upon reasonable request.
